# A practical method for multimodal registration and assessment of whole-brain disease burden using PET, MRI, and optical imaging

**DOI:** 10.1038/s41598-020-74459-1

**Published:** 2020-10-14

**Authors:** Matthew L. Scarpelli, Debbie R. Healey, Shwetal Mehta, Vikram D. Kodibagkar, Christopher C. Quarles

**Affiliations:** 1 Department of Neuroimaging, Barrow Neurological Institute, St. Joseph’s Hospital and Medical Center, Phoenix, AZ USA; 2 Department of Neurobiology, Barrow Neurological Institute, St. Joseph’s Hospital and Medical Center, Phoenix, AZ USA; 3 School of Biological and Health Systems Engineering, Arizona State University, Tempe, AZ USA

**Keywords:** Cancer imaging, Diseases of the nervous system

## Abstract

Many neurological diseases present with substantial genetic and phenotypic heterogeneity, making assessment of these diseases challenging. This has led to ineffective treatments, significant morbidity, and high mortality rates for patients with neurological diseases, including brain cancers and neurodegenerative disorders. Improved understanding of this heterogeneity is necessary if more effective treatments are to be developed. We describe a new method to measure phenotypic heterogeneity across the whole rodent brain at multiple spatial scales. The method involves co-registration and localized comparison of in vivo radiologic images (e.g. MRI, PET) with ex vivo optical reporter images (e.g. labeled cells, molecular targets, microvasculature) of optically cleared tissue slices. Ex vivo fluorescent images of optically cleared pathology slices are acquired with a preclinical in vivo optical imaging system across the entire rodent brain in under five minutes, making this methodology practical and feasible for most preclinical imaging labs. The methodology is applied in various examples demonstrating how it might be used to cross-validate and compare in vivo radiologic imaging with ex vivo optical imaging techniques for assessing hypoxia, microvasculature, and tumor growth.

## Introduction

Disease heterogeneity arises from spatial and temporal variations in genetic and phenotypic properties of tissue. This heterogeneity is often a barrier to effective treatment and can lead to variable responses, making selection of optimal therapy challenging^1–4^. This is particularly relevant for neurologic diseases, including brain cancers and neurodegenerative disorders, which are characterized by a high degree of heterogeneity^1,2,4–7^. For example, high grade gliomas can demonstrate highly variable blood flow, molecular expression levels, and invasive potential within and across tumors^3,8–10^. Further complicating treatment of neurologic disorders includes occurrence of disease at multiple locations within the brain, making localization and burden assessment difficult when relying on conventional pathologic assessments such as tissue biopsies. For example, subtypes of Alzheimer’s disease lead to neurofibrillary tangles and tau deposition in different brain regions^6,11^. Techniques for measuring disease burden across the whole-brain would enable improved understanding of these diseases and would facilitate selection of the appropriate systemic or localized therapy^12,13^.

Radiologic imaging techniques such as magnetic resonance imaging (MRI) or positron emission tomography (PET) offer means of non-invasively investigating disease phenotypes. A variety of PET tracers are under development for improving assessment of dementia, brain tumors, and other neurological conditions^14–18^. These techniques provide repeated longitudinal evaluation of whole brain physiology, making them crucial tools for assessing neurologic disease burden and disease heterogeneity in vivo^19^. Although these imaging modalities hold great potential, they have uncertainties (e.g. limited spatial resolution, non-specific uptake of contrast agents) that can diminish their reliability. Along these lines, measurements derived from radiologic images often include contribution from multiple physiologic quantities making clinical interpretation challenging. For example, T2 weighted MRI is routinely used clinically to identify regions of brain tumor infiltration; however, these images show increased signal in cancerous and non-cancerous (vasogenic edema) areas that leads to ambiguity^20^. Unfortunately, a translational barrier for many imaging techniques is lack of a clear link between imaging parameters and underlying biologic quantities^13,21–23^.

Ex vivo optical reporter imaging offers a more direct measure of underlying biology with a variety of labelling techniques available for imaging specific biologic processes (e.g. labeled cells, molecular targets, microvasculature). These ex vivo optical techniques can provide useful cross-validation of in vivo imaging techniques and help establish a relationship between imaging biomarkers and underlying biology quantities. However, conventional analyses involves optical imaging of thin histologic tissue sections only microns in thickness, which can be technically challenging to co-align with in vivo radiologic images due to differences in image scale and field of view. Nonetheless, some studies have successfully developed methods for acquiring and analyzing thin histologic slices across the whole rodent brain and subsequent registration to in vivo images^24^. These methods often rely on atlas-based registrations and or non-deformable registration algorithms to enable co-alignment between the ex vivo histologic slices and in vivo radiologic images^24,25^. Optical clearing techniques have enabled optical imaging of larger samples of excised tissue (e.g., whole rodent brain), which can simplify tissue slicing and registration with in vivo images^26,27^. However, imaging of optically cleared tissue typically requires specialized equipment, such as microscopes with long working distance objectives and or light sheet capabilities^10,27,28^. An additional challenge is that most ex vivo optical imaging techniques require relatively long acquisition times to acquire full brain coverage.

To overcome these limitations, we describe a practical methodology that integrates in vivo radiologic imaging with ex vivo optical reporter imaging of rodent brain tissue (rats and mice). The developed methodology involves co-registration and localized comparison of in vivo radiologic images (PET, MRI) and ex vivo optical reporter images, facilitating cross validation of in vivo and ex vivo modalities. The method incorporates multiple unique features to achieve relatively fast whole-brain coverage with multiple imaging modalities, including: (1) optical clearing of tissue, which removes the light penetration issues that often limit optical imaging to thin histologic sections, (2) integration of pathology slicing with ex vivo MRI scanning to enable in vivo-to-ex vivo image registration, and (3) utilization of a preclinical in vivo optical imaging system for rapid acquisition of fluorescent images covering entire sections of cleared tissue. In this study, an IVIS (PerkinElmer, Waltham, Massachusetts) is repurposed for optical imaging of the excised tissue. The IVIS offers a large field of view and rapid acquisition relative to conventional ex vivo optical imaging systems. This enables high throughput optical imaging of excised rodent brains.

Here the developed methodology is applied in rodent brain tumor models to demonstrate how it enables assessment of disease heterogeneity and facilitates development of radiologic imaging techniques for assessing whole-brain disease burden. The C6 and 9L cell lines are well-established preclinical brain tumor models. The C6 cell line is a rat glioma tumor that has been utilized in preclinical research since the 1960s and shares several genetic and histopathologic features with human glioblastoma tumors^29,30^. The 9L cell line is a rat gliosarcoma tumor that was developed in the 1970s and is widely utilized for studying therapeutic effects^29,31^. In the current study, 9L, C6, and patient derived xenograft (PDX) brain tumors were orthotopically implanted in rats and mice. The developed methodology is used to cross-validate the morphologic appearance of the various tumor cell lines on radiologic and ex vivo fluorescence images.

## Results and discussion

**Figure 1:**
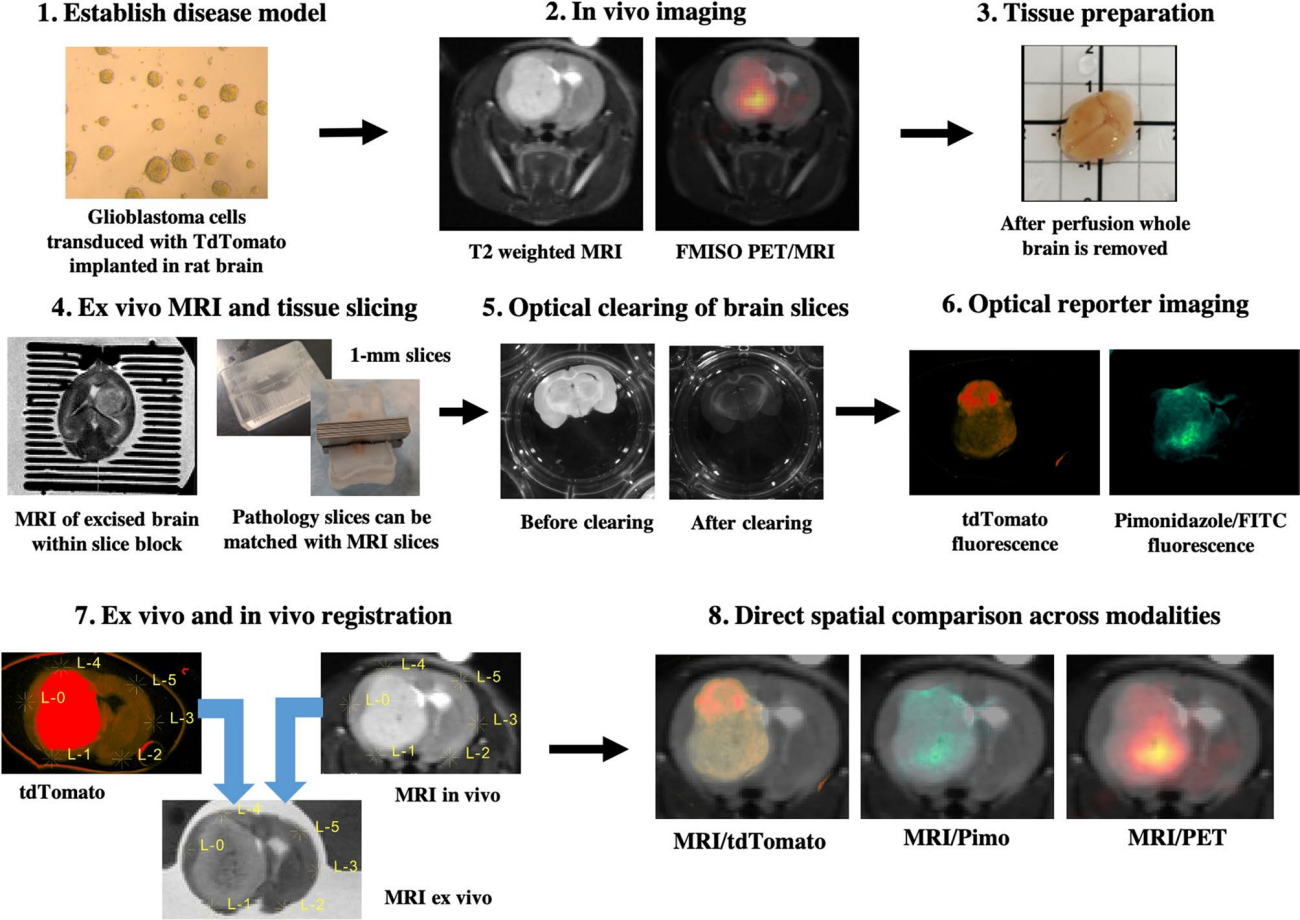
Overview of the developed methodology. Steps to acquire and co-align in vivo and ex vivo images of rodent brains. *FITC* fluorescein isothiocyanate, *FMISO* fluoromisonidazole, *MRI* magnetic resonance imaging, *PET* positron emission tomography, *Pimo* pimonidazole, *tdTomato* tandem dimer tomato-red fluorescent dye.

**Figure 2:**
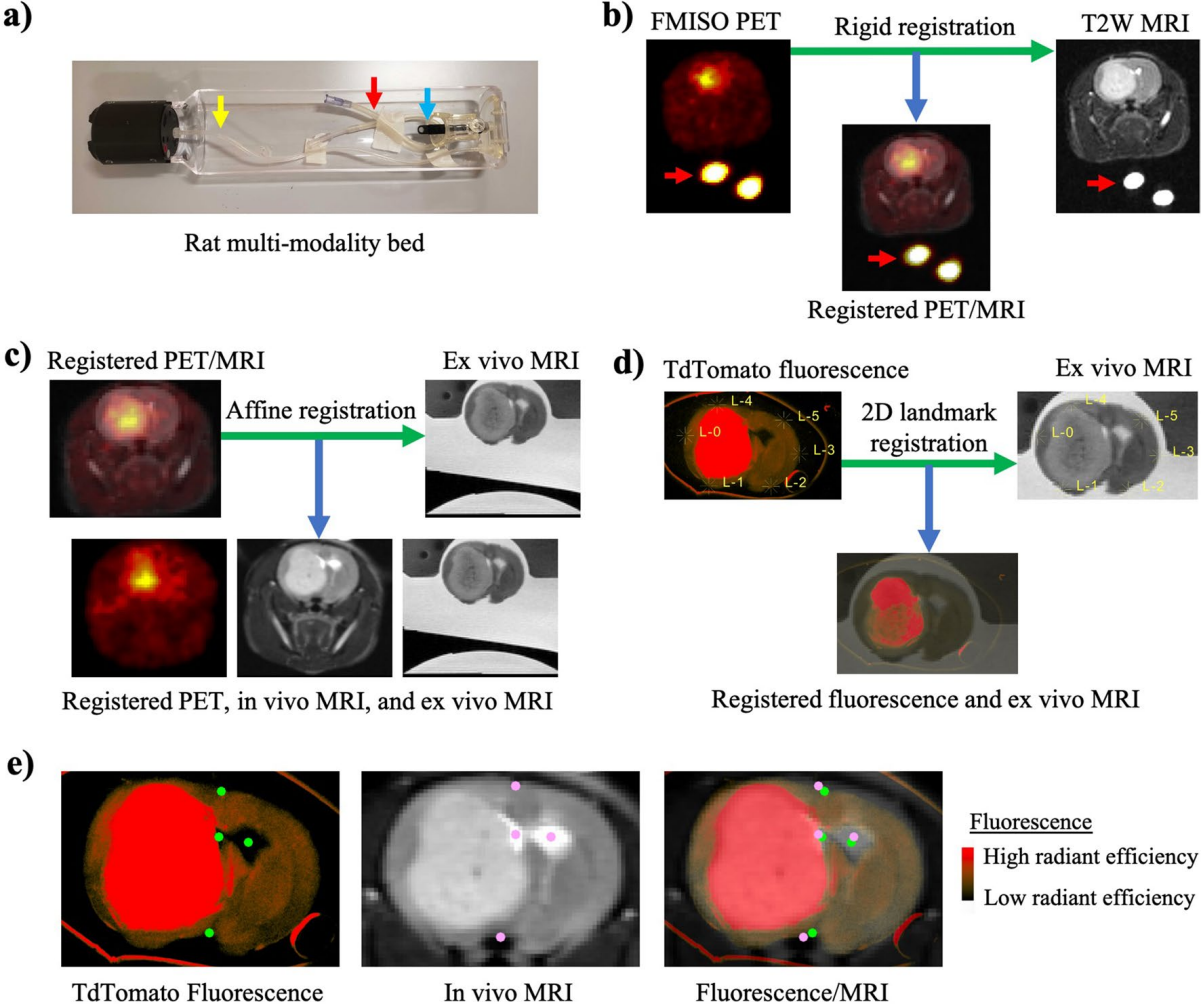
The multimodality image registration is a multi-step process with the goal of registering all images to the frame of reference of the ex vivo MRI. (**a**) Photograph of the rat multimodality bed (Bruker) that was utilized for imaging rats in both PET and MRI scanners. The anesthesia line (yellow arrow), registration phantom (red arrow), and nose cone (blue arrow) are identified for reference. The registration phantom was secured to the bottom of the bed and filled with PET tracer and water. (**b**) In vivo PET images were rigidly registered to in vivo MRI images using the registration phantom (red arrow) to guide the registration algorithm (i.e. a mask was placed on the registration phantom so that only this region was considered during the registration). (**c**) The registered in vivo images were rigidly registered to the ex vivo MR images. In this case a mask was placed around the brain on both the in vivo and ex vivo MR images so that only the brain voxels were considered during the registration. (**d**) Each pathology slice on the fluorescence images could be matched with the corresponding ex vivo MRI slice using the pathology slice block as a reference. Once matched, each fluorescence brain slice was registered to the corresponding ex vivo MRI slice using a 2D landmark registration (that includes a rigid registration plus uniform scaling). The landmarks utilized for the registration are shown and were consistently placed in the same six positions for each brain slice. (**e**) To evaluate the accuracy of the multimodality registration a new set of landmarks were manually placed on the in vivo MR images and at corresponding anatomic locations on the ex vivo fluorescence images. *MRI* magnetic resonance imaging, *PET* positron emission tomography, *FMISO* fluoromisonidazole, *tdTomato* tandem dimer tomato-red fluorescent dye.

The steps involved in the developed methodology are summarized in Fig. 1. The first step of establishing a disease model is specific to the particular application and disease being evaluated. Here, high-grade glioma cells are orthotopically implanted in rats and mice. After establishing the tumor model, in vivo imaging is performed (PET, MRI, etc.). The animal is then sacrificed, and the brain is removed. The brain is secured within a slice block and scanned ex vivo with MRI to enable in vivo-to-ex vivo registration. This is followed by slicing the brain into 1-mm pathology slices. The 1-mm pathology slices are optically cleared, and antibodies are added, if necessary, for optical imaging (e.g. anti-pimonidazole conjugated with fluorescein isothiocyanate (FITC) for detection of regional tumor hypoxia). Fluorescent imaging of the pathology slices is then performed using an IVIS. All images are registered to the ex vivo MRI so that they are within the same spatial frame of reference (Fig. 2a–d). In vivo MR and PET images are registered to ex vivo MR images using a affine registration (Fig. 2c). The fluorescent images of optically cleared brain slices are registered slice by slice to the ex vivo MRI slices using the pathology slice block as a reference (Fig. 2d).

The median difference in brain volume between the in vivo and ex vivo MR images was found to be − 5.3% (range − 11.9 to + 3.6%), indicating relatively little brain shrinkage. However, brain shrinkage could be greater if other tissue fixation methodologies are used, warranting use of the affine registration^32^. The accuracy of the in vivo MR to ex vivo MR image registration (Fig. 2c) was assessed by manually placing landmarks on anatomic regions defined by one author. A minimum of 40 landmarks per brain were placed across 7 rodent brains. After registration, the median distance between corresponding landmarks was found to be 250 μm with interquartile range of 120–410 μm (full range 10–910 μm). The accuracy of the ex vivo fluorescence to ex vivo MR image registration (Fig. 2d) was also assessed in a similar manner and the median distance between corresponding landmarks was found to be 390 μm with interquartile range of 230–540 μm (full range 70–870 μm). The accuracy of the entire multimodality image registration was also assessed (i.e. the accuracy of the registration between the in vivo MR images and the ex vivo fluorescence images). Landmarks were placed on in vivo MR images and ex vivo fluorescence images with landmark positions chosen so that they were clearly visible on both modalities (Fig. 2e). An average of 13 landmarks per brain were placed across 7 rodent brains. After registration, the median distance between corresponding landmarks was 400 μm with interquartile range of 240–580 μm (full range 40–980 μm). Given the spatial resolution of the in vivo medical images is typically on the order of a few hundred (MRI) or thousand (PET) micrometers, the accuracy of the developed multimodal registration method is sufficient for cross-validation of these imaging modalities. The accuracy of the current method is similar to the average registration error described by other multimodal image registration methods, which range from 200 to 400 μm^25,27^.

**Figure 3:**
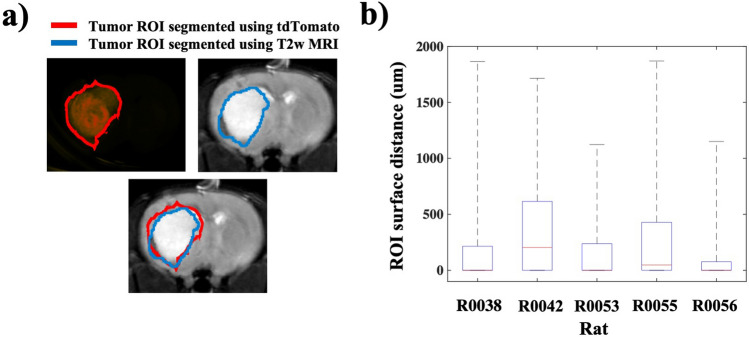
Surface distance measurements provide insight into spatial agreement between tumor ROIs defined using ex vivo fluorescent imaging and those defined using in vivo MR imaging. (**a**) Regions of interest (ROIs) were segmented on in vivo MR images and ex vivo fluorescence images. (**b**) The box plot shows distances between voxels on the surfaces of fluorescence- and MRI-defined ROIs for five 9L tumors. The median voxel surface distance value for each tumor is shown in red and the box whiskers show the minimum and maximum values. *MRI* magnetic resonance imaging, *tdTomato* tandem dimer tomato-red fluorescent dye.

**Figure 4:**
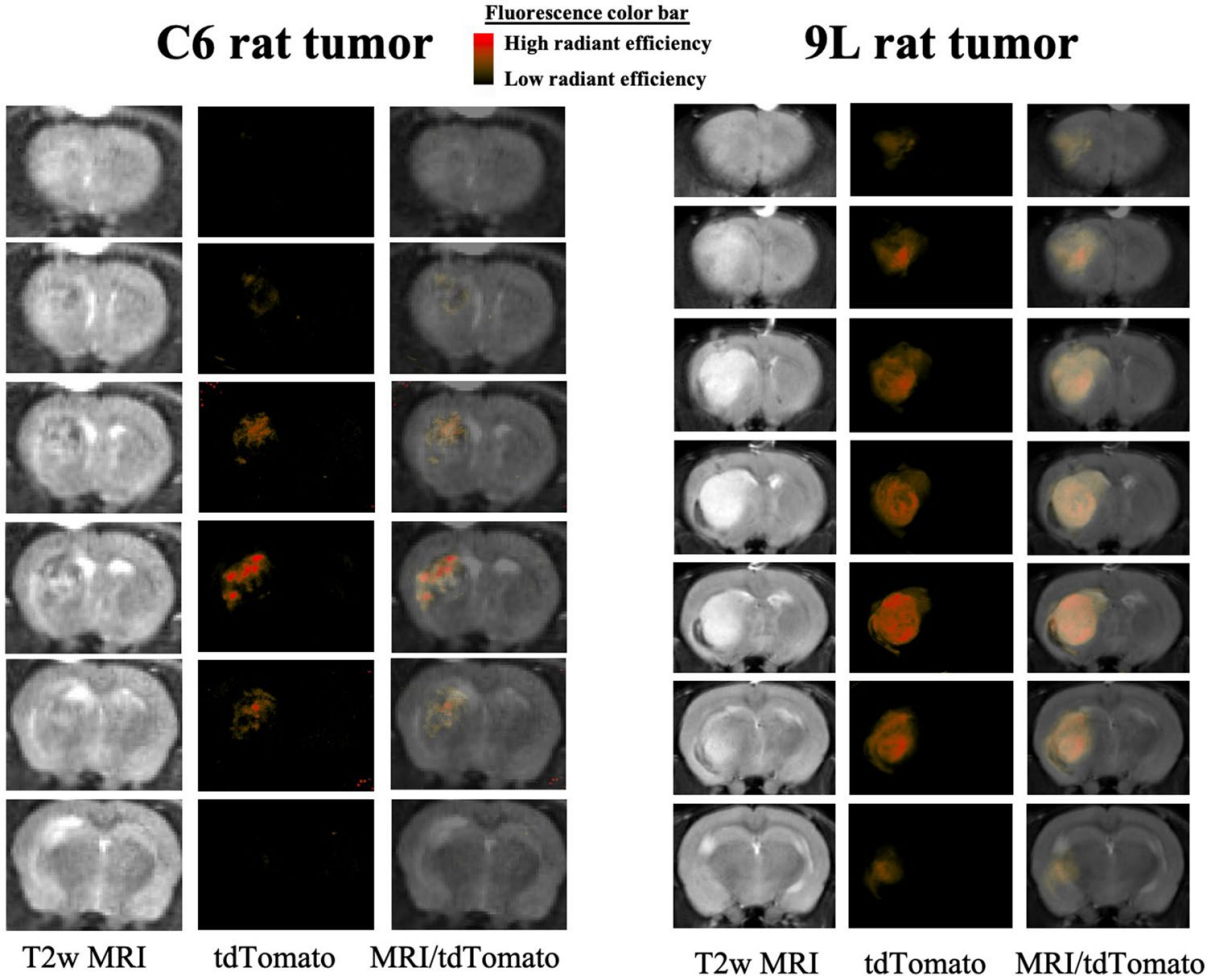
IVIS fluorescence images and T2w MR images of C6 and 9L tumors transduced with tdTomato. C6 tumors are known to be relatively diffuse and infiltrative whereas 9L exhibit sphere-like growth patterns with well-defined boundaries. *MRI* magnetic resonance imaging, *tdTomato* tandem dimer tomato-red fluorescent dye.

The well-circumscribed and clearly identifiable boundary of 9L tumors provided a useful model for cross validating the tumor boundary measured on MRI and ex vivo fluorescence images (Fig. 3a). Figure 3b summarizes surface distances between in vivo MRI and ex vivo fluorescence tumor segmentations for five 9L tumors after registering the images. The median surface distance value across all measurements was 34 μm with interquartile range 0–340 μm (full range 0–1870 μm). The mean surface distance value across all measurements was 220 μm with standard deviation of 320 μm. These results indicate the tumor margins identified on the in vivo MRI and ex vivo fluorescent images are within a few hundred microns of each other in most cases. For some voxels there was a large surface distance between the in vivo and ex vivo segmentations. This is evident from the maximum voxel surface distance values being between 1000 and 2000 μm for all tumor segmentations. These large surface distance values arise because the MRI has a lower sensitivity for detecting tumor regions than the ex vivo fluorescent imaging. The lower sensitivity is due primarily to partial volume effects and the indirect nature of T2 weighted MRI that measures tumor induced pathophysiologic changes (e.g. peritumoral edema) rather than tumor regions directly^20^. These effects are particularly evident at the edges of the tumor, where voxels may be only partially filled with tumor and therefore undetectable on the MRI. For example, the first and last coronal slices of the 9L tumor in Fig. 4, show slightly elevated fluorescence signal indicating tumor burden that is not evident on the MRI. The lower sensitivity of the MRI is also evident when considering that in every case the tumor volume defined on MRI was smaller than that defined on ex vivo fluorescence images (median tumor volumes were 13.4 mm^3^ and 20.4 mm^3^ for MRI and ex vivo fluorescence images, respectively). These results demonstrate how the developed methodology facilitates the validation of radiologic imaging techniques for identifying tumor burden and margins, which has implications for image-based radiotherapy and surgical planning^21,33^.

As an example application, the methodology is utilized to cross-validate the growth patterns of various tumor models on in vivo and ex vivo images. Figure 4 shows spatial patterns of glioblastoma growth in rats with C6 and 9L tumors. The C6 tumor consisted of alternating regions of high and low intensity on MR images with difficult to demarcate tumor boundaries. The C6 rat tumor was diffuse on IVIS fluorescent images and consisted of bands of high intensity extending from a central region. In contrast, the 9L tumor appeared as a centralized mass with clearly identifiable tumor boundaries on both MRI and ex vivo fluorescent images. These findings suggest C6 tumors may be more invasive than 9L tumors. This is in agreement with prior studies showing C6 rat tumors grow with a diffuse infiltrating border, whereas 9L rat tumors grow in a circumscribed pattern with little infiltration^29,34,35^.

**Figure 5:**
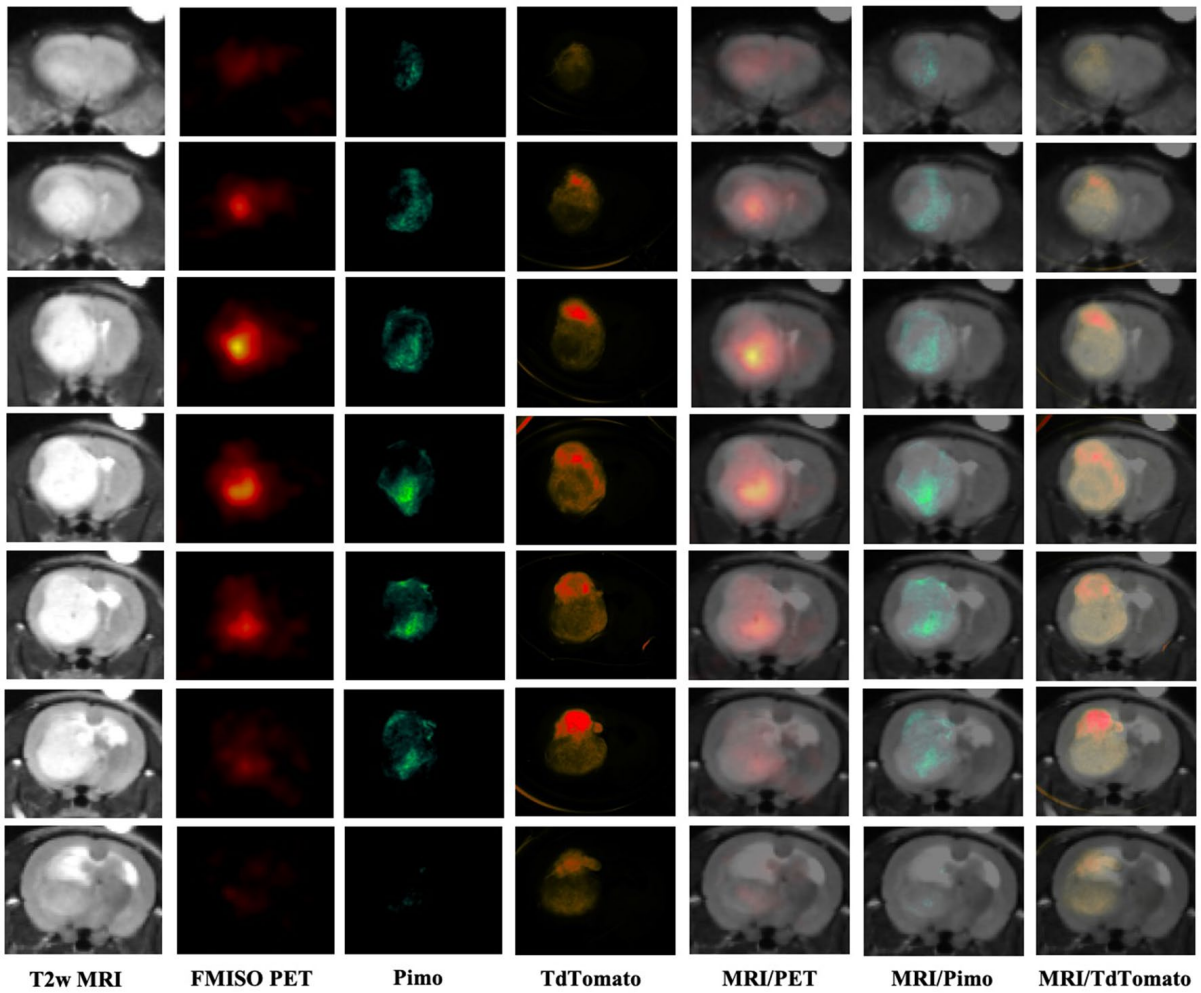
Coronal T2w MRI, PET, and IVIS fluorescence images of a rat brain with a 9L tumor. This example demonstrates how in vivo and ex vivo imaging modalities can be spatially aligned to validate radiologic measurements and characterize regional heterogeneity. *MRI* magnetic resonance imaging, *PET* positron emission tomography, *FMISO* fluoromisonidazole, *Pimo* pimonidazole, *tdTomato* tandem dimer tomato-red fluorescent dye.

The method can also be used to integrate in vivo radiologic imaging with ex vivo imaging of exogenously administered optical reporters (in this case, pimonidazole/antipimonidazole conjugated with FITC). Figure 5 and Supplementary Video S1 show ex vivo IVIS fluorescent images of brain slices compared with registered in vivo ^18^F-fluoromisonidazole (FMISO) PET and MRI slices. The T2 weighted MRI shows the implanted 9L tumor as a large mass within the right hemisphere of the brain. Both the PET and fluorescent pimonidazole images show high intensity in the inferior region of the tumor that is suggestive of hypoxia. Interestingly, the tandem dimer tomato-red fluorescent dye (tdTomato) fluorescent image of labelled tumor cells shows higher intensity in the superior region of the tumor where there is relatively low PET and fluorescence intensity. The reason for the intensity differences within the tumor may be due to tumor heterogeneity. For example, some tumor regions may have lower tumor cell density and consequently diminished tdTomato signal. Whereas other tumor regions may have higher tumor cell density and elevated tdTomato signal. This example demonstrates how the developed method might be used to characterize tumor subregions or “habitats”. Previous studies have indicated that measurement of tumor “habitats”, such as regions of hypoxia or normoxia and viable or nonviable tumor cells might be useful for predicting and assessing therapeutic response^3,8,36^.

**Figure 6:**
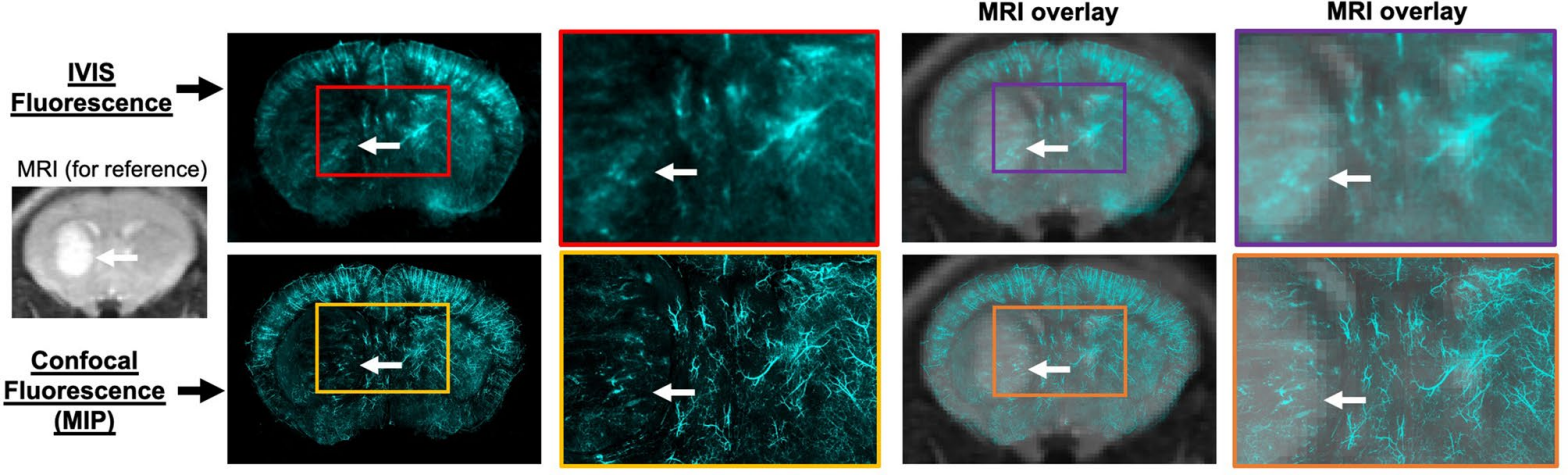
Coronal slices of mouse brain with a PDX GB126 tumor. Lectin fluorescence images were acquired using the IVIS and the confocal microscope (white arrow indicates tumor). This example demonstrates how optical images can be acquired across multiple scales, including mesoscopic (IVIS) and microscopic (confocal) for direct comparison with in vivo radiologic images. *MRI* magnetic resonance imaging, *MIP* maximum intensity projection.

Another application of the developed method is to integrate imaging techniques across multiple spatial scales. Although confocal fluorescent images take significantly longer to acquire than IVIS fluorescent images, the confocal microscope can be advantageous in cases were higher spatial resolution is needed. Figure 6 shows an example of this feature in which IVIS and confocal fluorescence images show lectin bound to blood vessels. The IVIS fluorescence image consists of high intensity regions corresponding to blood vessels; however, there is substantial blurring in the IVIS image due to overlapping of blood vessels through the 1 mm brain slice. The confocal fluorescence image enables visualization of even smaller blood vessels without blurring due to the 3D acquisition and higher spatial resolution. On both the IVIS and confocal images, the tumor exhibits low signal intensity as compared to the surrounding brain, indicating low tumor vascular volume fraction. The corresponding MRI slice is shown overlaid with the fluorescent images, providing a reference for the gross tumor volume.

Understanding how the micro and mesoscopic features of underlying tissue manifest on macroscopic radiologic images is important for clinical interpretation and/or validation of radiologic imaging techniques^13^. New radiologic imaging techniques are being developed for assessing neurological diseases such as amino acid PET (for brain tumors), amyloid- and tau- based PET (for neurodegenerative diseases), chemical exchange saturation transfer MRI (for brain tumors, cerebrovascular stroke, multiple sclerosis), and vessel architectural MRI (for vessel phenotyping)^16,37–39^. Our in vivo-to-ex vivo registration methodology offers a practical means to validate and optimize these radiologic imaging techniques by enabling direct spatial comparison between radiologic imaging features and specific optical reporters across the entire brain. This provides improvement over conventional methods of histologic validation, which typically sample only a small fraction of tissue using relatively thin slices.

The practical whole-brain assessment offered by our methodology also provides a more comprehensive technique for assessing spatial variations in disease pathogenesis. This is useful as neurologic diseases can be spatially heterogeneous and occur in multiple brain regions. Developing effective treatment strategies is going to require novel methods of measuring diseases across the brain that go beyond conventional pathologic measurements (e.g. biopsies and/or thin histologic sections). In this sense, our methodology might be useful, for example, in understanding why various subtypes of Alzheimer’s disease preferentially affect certain brain regions or why specific subregions of brain tumors do not respond to therapy^6^. For these types of analyses (i.e. analyses where image measurements are compared across brain regions), registration to a standard brain atlas could be beneficial to ensure consistency across studies. The developed methodology could be utilized for these purposes with an additional step for registering to an atlas. This could incorporate a workflow similar to that which has already been developed for registering MRI data to standard brain atlases^40^.

Methods for registering in vivo radiologic images with ex vivo optical images have been previously developed^24,25,27^. The advantages offered by the current method over other multimodality image registration methods are its relative simplicity and practicality. All image registrations utilized in our methodology can be performed using an open-source software package (3D Slicer) that is freely available for download (https://www.slicer.org/). Most preclinical imaging laboratories have an IVIS or comparable device for optical imaging of small animals. The developed methodology repurposes this widely available IVIS technology for imaging ex vivo samples of tissue, making this method practical for many preclinical imaging labs. The IVIS performs whole brain (rat and mouse) ex vivo optical imaging at far greater speeds and much larger field of views than other commercially available systems for optical imaging of excised tissue. The IVIS, with its rapid acquisition, facilitates high throughput fluorescence imaging of a large number of tissue slices in a relatively short amount of time (under 5 min to image slices from a whole rat brain). The large FOV also simplifies tissue preparation as brain slices can be imaged within a standard well plate using the IVIS. The in-plane spatial resolution of the IVIS is better than the in vivo medical imaging modalities and can serve as a useful tool for cross-validation in many applications (e.g. mapping the distribution of a fluorescent marker or tumor regions across the brain). The major disadvantages of utilizing the IVIS are that it is limited to 2D acquisitions and its in-plane spatial resolution is inferior to conventional optical imaging systems (e.g. confocal microscopes). In specific applications where higher spatial resolution or 3D acquisitions are needed (e.g. assessment of tissue microstructure, intracellular staining), conventional confocal microscopes could be utilized within the developed multimodality registration workflow but with significantly longer image acquisition times (Fig. 6).

In summary, we present a method that integrates the high sensitivity and specificity offered by optical reporter imaging with the translational potential of radiologic imaging. We showed how combining in vivo MRI with ex vivo imaging of fluorescent tumor cells provides insight into patterns of glioma growth and also the potential limitations of MRI for identifying tumor regions. We also showed how various sub-regions of disease might be identified using simultaneous measurement of hypoxia and tumor cells from MRI, PET, and ex vivo optical reporter imaging. The ability to integrate these complementary imaging technologies provides an opportunity to characterize multiple biological attributes important for disease pathogenesis across the entire rodent brain and to cross-validate measurements for investigating neurological diseases.

## Methods

### Animals and disease models

C6 and 9L glioblastoma cells were transduced with tdTomato to enable fluorescence imaging of the tumor cells ex vivo. C6 and 9L cells were harvested and resuspended in phosphate buffer saline (PBS) at a concentration of approximately 15 million cells/ml. A total of 4 µL of cell suspension was then orthotopically implanted in 5–9 weeks old Wistar (C6) and Fisher (9L) rats purchased from Charles River. For the GB126 PDX cell line, the cells were harvested and resuspended at a concentration of approximately 125 million cells/mL and 2 µL of cell suspension was orthotopically implanted into 5 weeks old NCRNU-M (CrTac:NCR-Foxn1 <nu> Homozygous) immunocompromised mice purchased from Taconic Biosciences (Rensselaer, New York). The PDX samples used for this research were provided by the Biobank Core Facility at St. Joseph’s Hospital and Medical Center and Barrow Neurological Institute. The samples were deidentified and conformed to the Biobank Institutional Review Board’s protocol. The St. Joseph Hospital and Medical Center’s Institutional Animal Care and Use Committee approved of all experimental procedures performed in this study and all animals were treated humanely in accordance with the Laboratory Animal Welfare Act.

### In vivo magnetic resonance imaging

MRI was performed 25–46 days after implantation of 9L tumor cells, 18–20 days after implantation of C6 tumor cells, and 41 days after implantation GB126 cells. MRI was performed with a 7-T Bruker Biospec preclinical MRI scanner. PET was performed using a Bruker Albira Si preclinical 3 ring PET scanner. PET was performed during the same imaging session as MRI. Animals were kept sedated when moving between PET and MRI scanners and remained positioned on the same Bruker multimodality rat bed. Each rodent’s teeth were secured within a bite bar that minimized motion of the head. In addition, the top of the head was secured to the bed using taped padding placed directly on top of the skull. Thus, the head is secured using a combination of the tape (from the top) and the bite bar (from the bottom), which mitigates any motion of the head. To enable accurate co-registration between the MRI and PET images, we placed a phantom fiducial filled with water and PET tracer beneath the rat in the multimodality bed to act as a landmark. During MRI and PET, the rats were kept sedated, with airflow of 1–1.5 mL/s with 1–3% isoflurane. MRI lasted approximately 15 min and included T2-weighted rapid acquisition with relaxation enhancement (RARE), with a repetition time of 6,500 ms, echo time of 50 ms, and a voxel size of 0.25 × 0.25 × 0.5 mm.

### Positron emission tomography

^18^F-fluoromisonidazole (FMISO) PET scans were acquired from 110 to 120 min post-injection of tracer with the brain positioned at the center of the field of view. An iterative algorithm was used for reconstruction with 12 iterations. Reconstructed images included corrections for scatter, deadtime, and decay of tracer.

### Perfusions

Following in vivo PET and MRI, the animals were sacrificed via transcardiac perfusion with 100 U/mL heparinized phosphate buffer (PB) to clear the blood from the system (25 mL for mice and 150 mL for rats). This procedure was followed by 4% paraformaldehyde (PFA) to fix the tissue (50 mL for mice and 300 mL for rats). One hour before perfusion, the rats were injected interperitoneally with pimonidazole at 60 mg/kg, for ex vivo detection of hypoxia. Ten minutes before perfusion, the mice were injected via the tail vein with 100 μL of lectin (DyLight 649–labeled Lycopersicon Esculentum Lectin, Vector Laboratories, Burlingame, California). Once perfusion was complete, the brain was dissected and immersed in 4% PFA for an additional 24–36 h to complete the fixation process. After immersion in PFA, the tissue was washed with 0.1-M PB and stored in 0.1-M PB.

### Ex vivo magnetic resonance imaging and tissue slicing

Ex vivo MRI was performed on whole rodent brains following the perfusions. During MRI, the whole brains were secured within a pathology slice block (Acrylic Brain Slicer Matrix, Zivic Instruments, Pittsburgh, Pennsylvania), which was placed within a cylindrical tube filled with 0.1-M PB. The MRI acquisition was set so that the brain slices were aligned parallel to the pathology slice block. Ex vivo MRI included the same parameters as the in vivo MRI. Immediately following MRI, the slice block with the brain was removed from the cylindrical tube, and the brain was sliced into 1-mm coronal slices. Each brain slice was then placed in 0.1-M PB in preparation for optical clearing. The brains were sliced into 1-mm slices because it was the thinnest size available in the acrylic MRI-compatible slice block.

### Optical clearing

A clear, unobstructed brain imaging cocktail (Cubic)-based protocol was utilized in clearing the tissue slices as previously described^28,41,42^, with slight modifications. Cubic is a passive clearing method consisting of two clearing reagents and a refractive index matching solution to help minimize light attenuation. Reagent-1 is prepared as a mixture of N,N,N′,N′-tetrakis(2-hydroxypropyl)ethylenediamine [30% (v/v)], urea [30% w/v)], Triton X-100 (17% (v/v)], and NaCl to a final concentration of 25 mM. The 1-mm brain slices were immersed in a 1:2 dilution of reagent-1 in water for 3–6 h at 37 °C then placed in undiluted reagent-1 for 6 days at 37 °C, with the reagent refreshed on days 2, 4, and 6. Reagent-2 is prepared as a mixture of sucrose [(52.4% (w/v)], urea [31.6% (w/v)] and 2,2′,2′-nitrilotriethanol [15% (v/v)]. On day 7, the samples were washed in 0.1-M PB and placed in a1:2 dilution of reagent-2 in 0.1-M PB for a minimum of 6 h to overnight at room temperature. This was followed by 2 days of undiluted Reagent-2 at 37 °C, with the reagent refreshed daily.

The samples that did not receive pimonidazole injections before perfusion were briefly washed in 0.1-M PB and placed in EasyIndex (a refractive index matching solution from LifeCanvas Technologies, Cambridge, Massachusetts) for 2–24 h at 37 °C. The samples that received pimonidazole were washed in 0.1-M PB and incubated at 4 °C for 3 days in 0.1-M PB with 0.1% triton-X and a 1:50 dilution of mouse IgG1 antipimonidazole monoclonal antibody conjugated with FITC (HP FITC Mab-1, Hypoxyprobe, Burlington, Massachusetts). After the 3-day incubation, the unbound antibody was washed from the tissue slice with 0.1-M PB at room temperature, and then, the slice was placed in EasyIndex (LifeCanvas Technologies).

### Fluorescence imaging

Optically cleared brain slices were imaged using either the IVIS Spectrum (PerkinElmer, Waltham, Massachusetts) for mesoscopic resolution or a confocal fluorescence microscope (Leica, Wetzlar, Germany) for microscopic resolution. For IVIS imaging, each set of brain slices were placed in a 12-well tissue culture plate filled with EasyIndex (LifeCanvas Technologies). An excitation wavelength of 570 nm and an emission wavelength of 620 nm was used for imaging the tdTomato protein. An excitation wavelength of 500 nm and an emission wavelength of 540 nm were used for imaging anti-Pimo FITC. For imaging lectin, an excitation wavelength of 640 nm and an emission wavelength of 680 nm were used. All IVIS imaging consisted of a 2D 60-s acquisition with a bin size of 1 (corresponding to 34.4 μm pixels), F/Stop of 8, and field of view of 6.6 cm × 6.6 cm. Two acquisitions per fluorescent probe were required to ensure the field of view covered the entire 12-well plate.

Confocal fluorescence imaging was performed using a Leica (Wetzlar, Germany) SP8 White Light Laser Confocal Microscope. The mouse brain slices were mounted on microscope slides in EasyIndex (LifeCanvas Technologies) for imaging. The brain slices perfused with lectin were imaged using a white light laser with an excitation of 650 nm and emission bandwidth of 660–700 nm. The in-plane pixel size was 3.56 × 3.56 μm, and the z-step size was 30 μm. The field of view covered the entire brain slice (9 mm × 12 mm × 1 mm). The total imaging time was approximately 60 min per brain slice. The resulting z-stack of confocal images was converted to a maximum intensity projection for visualization and analysis.

### Image registration

All image registrations were performed by one author using 3D Slicer v4.10.1 (Cambridge, Massachusetts). All rigid and affine registrations were performed using 3D Slicer’s General Registration (BRAINS) module that included an objective function based on mutual information^43^. Prior to performing rigid and affine registrations an initialization of the two images was performed using 3D Slicer’s interactive Transforms module. This initialization included manual translation and rotation of the images so that they were closely aligned prior to the rigid registration. In vivo PET and MRI images were rigidly registered using the phantom placed within the Bruker multimodality rat bed (i.e. an ROI was segmented on the phantom so that only it was considered during the registration). The phantom consisted of an approximately 8 cm flexible tube filled with water and PET tracer. The phantom was bent into a loop and taped flat to the bottom of the rat holder beneath the rat’s head to enable accurate co-registration (Fig. 2a,b). In vivo MR images were registered to ex vivo MR images using an affine registration (with 12 degrees of freedom) to account for any brain shrinkage when going from in vivo to ex vivo. The resulting transformation was also applied to register the transformed PET to the ex vivo MRI (Fig. 2c).

The fluorescence images of optically cleared brain slices were registered slice by slice to the ex vivo MRI slices using the pathology slice block as a reference. The pathology slice block enabled matching of the MRI slices with the pathology slices. Once the slices were matched a 2D landmark-based registration was performed to register each slice. The registration involved manual placement of six points (i.e. landmarks) along the edges of the brain in each image (Fig. 2d). 3D Slicer’s Landmark Registration Module was used to calculate the geometric transform that most closely aligned the corresponding landmarks placed within the two images^44^. The geometric transform included a rigid registration plus uniform scaling (to account for shrinkage/expansion of the cleared tissue).

### Image analysis

To evaluate the ability of MRI to identify the tumor margins, one author defined regions of interest (ROIs) around the 9L tumors on both the in vivo MR images and the ex vivo tdTomato fluorescence images. The tumor ROI segmented on the MR images was manually defined by segmenting tumor regions with hyperintensity (excluding the hyperintense ventricles). The tumor ROI for tdTomato fluorescence images was defined using a semi-automatic threshold-based technique. The intensity threshold was selected individually for each brain slice and included selection of the lowest threshold value that led to no contralateral brain voxels being included in the tumor ROI. The surface distance between the in vivo and ex vivo ROIs was calculated for each voxel on the surface of the tumor ROI. The surface distance was calculated by determining the minimum distance between a given surface voxel and any voxel on the surface of the other ROI. The metric was summarized for each tumor by the median surface distance across all voxels on the surfaces of the ROIs. For surface distance calculations, the in vivo MR images were upsampled to match the voxel size of the ex vivo fluorescence images.

## Supplementary information

Supplementary file 1

Supplementary file 2

## Data Availability

The datasets generated and analyzed during the current study are available from the corresponding author on reasonable request.
