# Supplementary material for: A practical method for multimodal registration and assessment of whole-brain disease burden using PET, MRI, and optical imaging
Source: Sci Rep. 2020 Oct 14;10:17324. doi: 10.1038/s41598-020-74459-1 (PMC7560610; doi:10.1038/s41598-020-74459-1)
Supplement: Supplementary file 1 [file 41598_2020_74459_MOESM1_ESM.docx]

**A practical method for multimodal registration and assessment of whole-brain disease burden using PET, MRI, and optical imaging**

Matthew L. Scarpelli, PhD^1^ Debbie R. Healey, BS^1^ Shwetal Mehta, PhD^2^ Vikram Kodibagkar, PhD^3^ Chad C. Quarles, PhD^1*^

**Supplementary Video S1*.*** Three-dimensional reconstruction of the rat brain with a 9L tumor that is shown in Figure 5 of the manuscript. The in vivo positron emission tomography (PET) and magnetic resonance images (MRIs) were registered to the ex vivo fluorescence images to enable a localized comparison of tumor regions across modalities. The fluoromisonidazole (FMISO) PET and pimonidazole (pimo) fluorescence images highlight regions of hypoxia whereas the tdTomato fluorescence image highlights labelled tumor cells. The fluorescence images were acquired ex vivo using an in vivo imaging system (IVIS), which has multiple advantages over traditional methods of ex vivo fluorescence imaging, including rapid acquisition, accessibility, and ease of use. The spatial resolution of the IVIS is in the mesoscopic range (~30 μm) and if higher resolutions are needed then confocal fluorescence microscopy must be used. *Used with permission from Barrow Neurological Institute, Phoenix, Arizona.*
